# Vaccine-hesitant individuals accumulate additional COVID-19 risk due to divergent perception and behaviors related to SARS-CoV-2 testing: a population-based, cross-sectional study

**DOI:** 10.1007/s15010-022-01947-z

**Published:** 2022-11-10

**Authors:** Paul R. Wratil, Katharina Kotter, Marie L. Bischof, Sophie Hollerbach, Elif Apak, Anna-Lena Kalteis, Tamara Nayeli-Pflantz, Lars Kaderali, Kristina Adorjan, Oliver T. Keppler

**Affiliations:** 1 Max von Pettenkofer Institute and Gene Center, Virology, National Reference Center for Retroviruses, LMU München, Munich, Germany; 2 German Center for Infection Research (DZIF), Partner Site Munich, Munich, Germany; 3 Institute of Bioinformatics, University Medicine Greifswald, Greifswald, Germany; 4 Department of Psychiatry and Psychotherapy, University Hospital, LMU München, Nußbaumstraße 7, 80336 Munich, Germany; 5 Max von Pettenkofer Institute and Gene Center, Virology, LMU München, Feodor-Lynen-Str. 23, 81377 Munich, Germany; 6 Faculty of Medicine, Max von Pettenkofer Institute and Gene Center, Virology, LMU München, Pettenkoferstr. 9a, 80336 Munich, Germany

**Keywords:** SARS-CoV-2, COVID-19, Surveillance, Rapid antigen test, PCR test, Vaccination

## Abstract

**Purpose:**

To investigate the perception of SARS-CoV-2 detection methods, information sources, and opinions on appropriate behavior after receiving negative or positive test results.

**Methods:**

In a questionnaire-based, cross-sectional study conducted between September 1 and November 17, 2021, epidemiological, behavioral, and COVID-19-related data were acquired from the public in Munich, Germany.

**Results:**

Most of the 1388 participants obtained information from online media (82.8%) as well as state and federal authorities (80.3%). 93.4% believed in the accuracy of SARS-CoV-2 PCR testing and 41.2% in the accuracy of rapid antigen tests (RATs). However, RATs were preferred for testing (59.1%) over PCR (51.1%). 24.0% of all individuals were willing to ignore hygiene measures and 76.9% were less afraid of SARS-CoV-2 transmission after receiving a negative PCR test (5.9% and 48.8% in case of a negative RAT). 28.8% reported not to self-isolate after receiving a positive RAT. Multivariate analyses revealed that non-vaccinated individuals relied less on information from governmental authorities (*p* = 0.0004) and more on social media (*p* = 0.0216), disbelieved in the accuracy of the PCR test (*p* ≤ 0.0001) while displaying strong preference towards using RATs (*p* ≤ 0.0001), were more willing to abandon pandemic-related hygiene measures (*p* ≤ 0.0001), less afraid of transmitting SARS-CoV-2 after a negative RAT (*p* ≤ 0.0001), and less likely to isolate after a positive RAT (*p* ≤ 0.0001).

**Conclusion:**

Insights into preferred information sources as well as perception, preferences, and behavior related to SARS-CoV-2 testing and hygiene measures are key to refining public health information and surveillance campaigns. Non-vaccinated individuals’ divergent believes and behaviors possibly increase their COVID-19 risk.

**Supplementary Information:**

The online version contains supplementary material available at 10.1007/s15010-022-01947-z .

## Introduction

The Coronavirus Disease 2019 (COVID-19) caused by the Severe Acute Respiratory Syndrome Coronavirus 2 (SARS-CoV-2) rapidly evolved to a pandemic in early 2020. COVID-19 is a major threat to global health and, to this date, dictates policymaking around the world. Based on experiences with the SARS-CoV-2 variants of concern (VoCs), especially Delta and Omicron, it is conceivable that novel virus variants with increased pathogenicity as well as enhanced transmissibility and immune escape will emerge in the future, potentially diminishing the success of vaccination [1]. Therefore, effective surveillance of SARS-CoV-2 infections will remain critical to control the ongoing COVID-19 pandemic. This requires both accurate and scalable testing methods as well as a public that is well informed about these tests and willing to perform them. Especially non-vaccinated individuals should be knowledgeable about the quality and usefulness of SARS-CoV-2 detection methods as they are more prone to SARS-CoV-2 infection and transmission as well as to severe COVID-19 [2–4].

The gold standard for the diagnosis of acute SARS-CoV-2 infections is the detection of viral RNA via nucleic acid amplification from nasopharyngeal swabs (PCR test). Due to their capability to detect even low viral loads (limit of detection < 1000 copies/mL) [5–7], PCR tests have a high diagnostic sensitivity and can identify infections already three days before symptom onset [8]. The amplification of several non-*spike* gene-targeted sequences in PCR tests assures high sensitivity also for the detection of VoCs carrying numerous *spike* mutations [9, 10]. There are, however, several caveats for the use of PCR tests for SARS-CoV-2 surveillance, among others, the long turn-around time of at least several hours, but often one or two days, in routine diagnostics. Performing PCR assays requires well-equipped laboratories with trained personnel and is relatively expensive.

A frequently employed alternative to PCR testing is the use of rapid antigen tests (RATs) that detect the viral nucleocapsid protein in nasopharyngeal or oral swabs. RATs are simple to use diagnostic devices, typically based on lateral flow technology. One advantage of such RATs is their availability at low cost and in high quantities. RATs do not require special laboratory equipment and can be performed both by healthcare professionals and as self-tests. Moreover, the time-to-result of RATs is comparably short (approximately 15–30 min). Therefore, these assays are commonly used for point-of-care testing. A disadvantage of RATs, however, is their low diagnostic and clinical sensitivity compared to PCR tests [11–13], and their enormous performance heterogeneity [14, 15]. Particularly, the sensitivity to detect the nowadays predominant SARS-CoV-2 VoC omicron appears to be dismal using RATs [16–18], challenging the usefulness of RATs for COVID-19 diagnostics and surveillance, in particular in high-risk settings.

Detailed information on the pros and cons of the two predominant COVID-19 detection methods is important, but it is unclear to what extend the general public is knowledgeable about the complex differences between PCR and RAT. To address this, we investigated the perception of SARS-CoV-2 testing methods among citizens in Munich, Germany, in a cross-sectional study including 1388 participants grouped by age, gender, education, native language as well as vaccination status. We analyzed which media sources were used for gathering general information on SARS-CoV-2 testing, how the accuracy of RATs and PCR tests was perceived, as well as which detection method was preferred by the participants. Additionally, we surveyed the opinion on appropriate behavior after receiving either negative or positive SARS-CoV-2 test results.

## Materials and methods

### Study design, setting, and participants

Between September 1 and November 17, 2021, we invited the public of Munich, Germany, to participate in a cross-sectional study. Utilizing handouts and posters the study was advertised in restaurants, a shopping mall, at COVID-19 test centers, a quaternary care university hospital (LMU Klinikum) and on different sites of the Ludwig Maximilian University of Munich (LMU München). Additionally, we recruited participants for the study using banners in subways during the month of October 2021. Only individuals who reported being of legal age (18 years and above) were included in the study.

### Data collection

Participants anonymously answered an online questionnaire comprised of 42 items. Herein, demographical, and epidemiological data were assessed, including participants’ gender, age, level of education, profession, and vaccination status, as well as reasons against vaccination in non-vaccinated individuals. Furthermore, specific questions about SARS-CoV-2 testing via PCR or RAT were answered, among others which media sources were used for gathering information on testing, beliefs in the accuracy of the different testing methods, preferences between testing methods and opinions of appropriate behaviors after receiving negative or positive test results. The questionnaire was available in five different languages, namely English, German, Italian, Spanish and Turkish. The English version of the study questionnaire is available in the supplementary information (Supplementary Table 1). Only data obtained from completed questionnaires were used for further evaluation.

### Statistical analysis

Data were analyzed in R version 4.1.2 (www.r-project.org). *p*-values on pair-wise comparisons were calculated using Fisher’s exact test—in case of multiple testing—with Holm’s multiple testing correction for categorical variables and Wilcoxon rank sum test with continuity correction for ordinal scaled data as indicated. Multivariate analyses were performed using logistic regressions, with the responses to specific questions, e.g., agreement or disagreement to a statement, as dependent and gender, language, age group, level of education, as well as healthcare worker and vaccination status as independent variables. Significance in statistical tests was assigned the following designation: *p ≤ 0.05, **p ≤ 0.01, ***p ≤ 0.001, ****p ≤ 0.0001.

## Results

### Study population

**Fig. 1:**
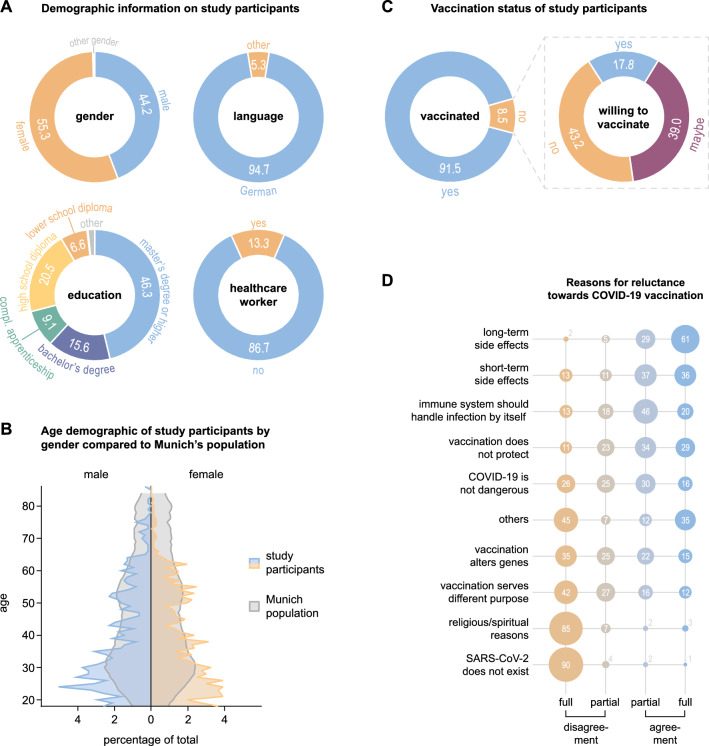
Overview of the study population. **A** Demographic information on the study participants. Information is shown as percentage of all 1388 participants. **B** Age demographic of study participants by gender (blue and orange shaded) compared to Munich’s population in 2021 (gray shaded) [19]. **C** Percentage of COVID-19-vaccinated individuals among all participants (left) and percentages of non-vaccinated individuals willing to vaccinate (right). **D** Agreement and disagreement (partial or full) to different reasons for reluctance towards vaccination among those 97 participants indicating to be not or maybe willing to get vaccinated. Absolute numbers of participants giving certain answers are displayed

1388 individuals (approximately 0.1% of Munich’s population) participated in the study and completed the questionnaire, of whom 55.3% (767/1388) were female, 44.2% (614/1388) were male, and 0.1% (7/1388) were of other gender (Fig. 1A). The majority of participants answered the questionnaire in German language, i.e., 94.7% (1315/1388, Fig. 1A). Asking for their highest level of education, we found that 46.3% (642/1388) of all participants were holding a master’s or a higher academic degree, 15.6% (216/1388) had a bachelor’s degree, 9.1% (127/1388) completed an apprenticeship, 20.5% (285/1388) had a high school, and 6.6% (92/1388) a lower school diploma (Fig. 1A). Among all participants, there were 13.3% (184/1388) healthcare workers, e.g., physicians or nurses (Fig. 1A).

Comparing the age demographic of the participants to Munich’s population in 2021 [19], we found that, albeit being similar, a larger fraction of young individuals and less elderly participated in the study (Fig. 1B). Categorizing all participants into four different age groups, 52.4% (728/1388) were between 18 and 35, 23.3% (324/1388) between 36 and 50, 20.5% (284/1388) between 51 and 65, and 3.7% (52/1388) more than 65 years old.

### COVID-19 vaccination status and vaccine hesitancy

8.5% (118/1388) of all participants reported to be not vaccinated against COVID-19 (Fig. 1C). Among those 17.8% (21/118) stated that they were planning to become vaccinated, whereas 39.0% (46/118) were uncertain about getting vaccinated, and 43.2% (51/118) reported being unwilling to get vaccinated (Fig. 1C). No major differences in vaccination rates and rates of individuals reluctant to become vaccinated could be observed comparing the different participant subgroups (Supplementary Fig. 1), except for healthcare workers who reported significantly higher vaccination rates (95.7%, 95% CI 91.7–97.8) compared to others (90.9%, 95% CI 89.1–92.4, *p, Supplementary Fig. 1C). The most frequently given reasons for uncertainty or unwillingness regarding COVID-19 vaccination were fear of long-term and/or short-term side effects (90/97 and 73/97 participants agreed fully or partially, respectively) followed by beliefs that the immune system should be capable of handling a SARS-CoV-2 infection by itself (66/97 participants agreed) and that vaccines are not protective against COVID-19 (63/97 participants agreed, Fig. 1D).

Performing subgroup analysis (Supplementary Fig. 2, 3), we observed that females, who were reluctant to get vaccinated, more often indicated to be afraid of possible long-term side effects (98.3%, 95% CI 90.9–99.7) than males (84.2%, 95% CI 69.6–92.6, *p, Supplementary Fig. 3B), whereas males more often agreed that they were afraid of the vaccination serving another purpose than protection against a virus (42.1%, 95% CI 27.9–57.8) compared to females (20.7%, 95% CI 12.3–32.8, *p, Supplementary Fig. 3B). Additionally, individuals with a lower school diploma as their highest degree of education more often indicated that they feared the vaccination could alter their genes (85.7%, 95% CI 48.7–97.4) than participants holding a master’s or a higher academic degree (20.5%, 95% CI 10.8–35.5, *p, Supplementary Fig. 3F).

### Preferred sources for information on testing for acute SARS-CoV-2 infection

**Fig. 2:**
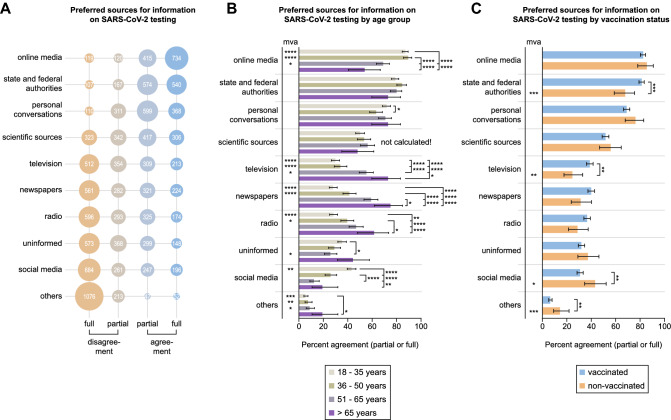
Information sources for SARS-CoV-2 testing. **A** Agreement and disagreement (partial or full) on preferring different information sources for SARS-CoV-2 testing among all 1388 participants. Absolute numbers of participants giving certain answers are displayed. **B**, **C** Percentages of all participants agreeing (partially or fully) on gathering information on testing using different sources by age group (**B**) and vaccination status (**C**). Error bars indicate 95% confidence intervals. Asterisks next to brackets indicate statistical significance between groups calculated using Fisher’s exact test (**C**) or Fisher’s exact test with Holm’s testing correction (**B**). Asterisks below “mva” indicate statistical significance in multivariate analysis. If no asterisks are given, no statistical significance was detected. mva—multivariate analysis

We asked the participants which type of media they most frequently use to inform themselves about SARS-CoV-2 testing. The information sources most participants agreed (fully or partially) on using were online media (82.8%, 1149/1388) followed by information from state and federal authorities (80.3%, 1114/1388, Fig. 2A).

Individuals from younger age groups (18–35 and 36–50 years) agreed significantly more often to obtain information from online media (87.2%, 95% CI 84.6–89.5, and 89.5%, 95% CI 85.7–92.4, respectively) than participants from older age groups both in direct comparison (****p) and in multivariate analysis (****p). Participants between 18 and 35 years more frequently indicated to use social media for gathering information on testing (43.1%, 95% CI 39.6–46.8) compared to other age groups (**p–****p) and in multivariate analysis (**p, Fig. 2B). On the contrary, there was a trend towards using television, radio, and newspapers less frequently in younger than in older age groups that was statistically significant in most cases, both in direct comparison and in multivariate analysis (Fig. 2B).

Interestingly, non-vaccinated individuals agreed significantly less often to obtain information on SARS-CoV-2 testing from state and federal authorities (67.8%, 95% CI 58.9–75.5) as well as television (24.5%, 95% CI 17.7–33.1) than vaccinated participants (81.4%, 95% CI 80.3–84.5, ***p and 38.8%, 95% CI 36.2–41.5, **p, respectively) and in multivariate analysis (***p and **p, Fig. 2C). However, non-vaccinated individuals indicated to rely on social media more frequently as an information source (43.2%, 95% CI 34.6–52.2) than vaccinated individuals (30.9%, 95% CI 28.4–33.5, **p) and in multivariate analysis (*p, Fig. 2C).

The results from other subgroup analyses are depicted in Supplementary Fig. 4. We observed that males agreed less frequently on gathering information via state and federal authorities (75.1%, 95% CI 71.5–78.3), personal conversations with others (65.6%, 95% CI 45.9–53.8) and the radio (32.9%, 95% CI 29.3–36.7) compared to females (84.5%, 95% CI 81.8–86.9, ****p, 72.8%, 95% CI 69.5–75.8, **p, and 38.3, 95% CI 35.0–41.8, *p, respectively) as well as in multivariate analysis (****p, **p, *p, Supplementary Fig. 4A). Individuals who answered the questionnaire in languages other than German reported significantly more often to inform themselves using media by state and federal authorities (90.4%, 95% CI 81.5–95.3) and scientific sources (69.9%, 95% CI 58.6–79.2) compared to German speakers (79.7%, 95% CI 77.4–81.8, and 51.1%, 95% CI 48.4–53.8, *p, **p) and in multivariate analysis (*p, ***p, Supplementary Fig. 4B). Multivariate statistical analysis also revealed that participants with a lower school diploma as their highest academic degree relied less on online media (*p) and scientific sources (**p) but stated more frequently to use the radio (**p) or stay uninformed (*p, Supplementary Fig. 4C). Furthermore, healthcare workers (HCW) indicated to gather information on COVID-19 testing more regularly using scientific sources (73.4%, 95% CI 66.6–79.2) compared to others (48.8%, 95% CI 46.0–51.7, ****p) and in multivariate statistical analysis (****p).

### Trust in accuracy and personal preferences comparing different SARS-CoV-2 detection methods

**Fig. 3:**
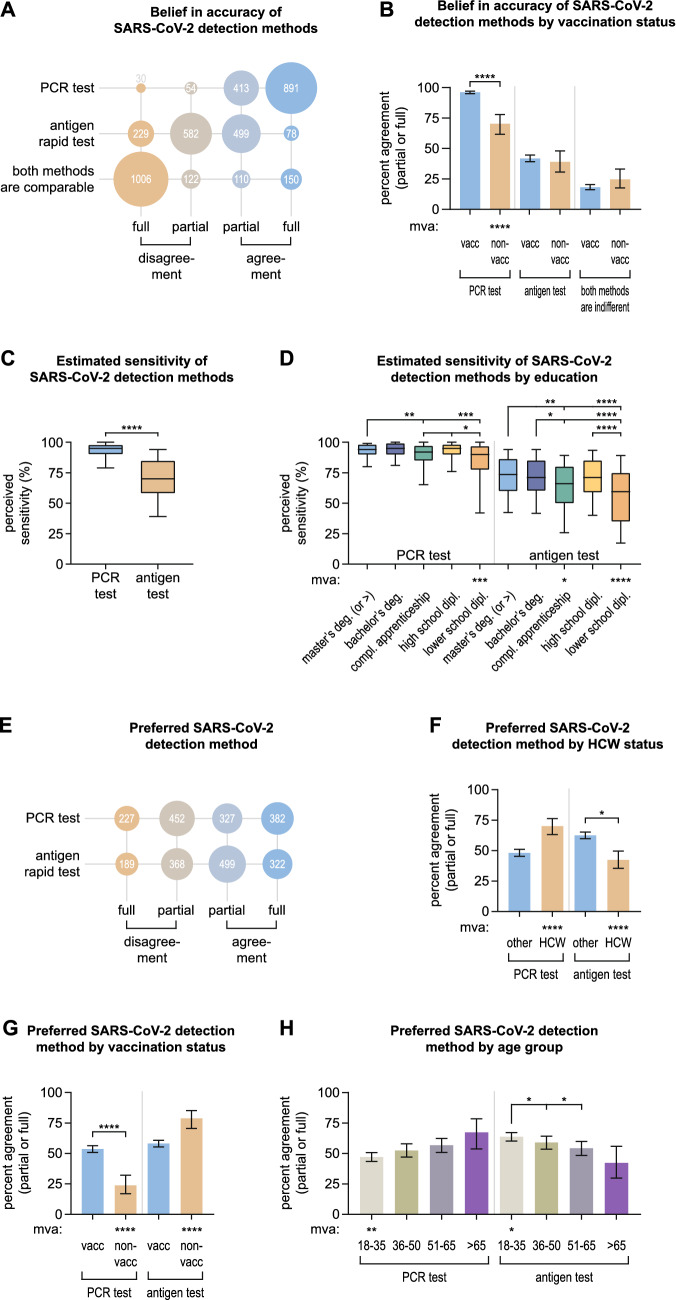
Belief in accuracy and personal preferences comparing different SARS-CoV-2 detection methods. **A** Agreement and disagreement (partial and full) on believing in the accuracy of the PCR and RAT and the statement of being unaware of differences between the two SARS-CoV-2 detection methods among all 1388 participants. **B** Percentages of all participants agreeing (partially or fully) on believing in the accuracy of the two testing methods or considering them indifferent by vaccination status. **C** Comparison of the estimated sensitivities of both testing methods among all participants. **D** Estimated sensitivities by participants’ highest degree of education. **E** Agreement and disagreement (partial and full) for preferring the PCR and RAT for COVID-19 testing among all participants. **F**–**H** Percentages of all participants agreeing (partially or fully) on preferring either of the two testing methods by healthcare worker status (**F**), vaccination status (**G**), and age group (**H**). Absolute numbers of participants giving certain answers are displayed in (**A**, **E**). Error bars in (**B**, **F**–**H**) indicate 95% confidence intervals. Box plots in (**C**, **D**) depict medians, bounds between upper and lower quartiles, and whiskers between the 10th and 90th percentiles. Asterisks above brackets indicate statistical significance between groups calculated with Fisher’s exact test (**B**, **F**, **G**), Wilcoxon rank sum test with continuity correction (**C**, **D**), and Fisher’s exact test with Holm’s testing correction (**H**). Asterisks next to “mva” indicate statistical significance in multivariate analysis. If no asterisks are given, no statistical significance was detected. compl.—completed, deg.—degree, dipl.—diploma, HCW—healthcare worker, mva—multivariate analysis, non-vacc—non-vaccinated, vacc—vaccinated

Next, we sought to assess the trust in the diagnostic accuracy of the PCR and the RAT and personal preferences in using one or the other SARS-CoV-2 detection method. We first asked the participants whether they were convinced of the accuracy of any of the two assays, and whether they knew about differences between the PCR and the RAT. 93.4% (1304/1388) individuals agreed (fully or partially) that they believed in the accuracy of the PCR test, whereas only 41.2% (577/1388) stated to believe in the accuracy of the RAT (Fig. 3A). 18.7% (260/1388) agreed that both methods were comparable (Fig. 3A). Non-vaccinated individuals indicated significantly less frequently to believe in the accuracy of PCR testing (70.3%, 95% CI 61.2–77.8) compared to vaccinated persons (96.1%, 95% CI 94.9–97.1, ****p) and in multivariate analysis (****p, Fig. 3B). Results from additional subgroup analyses are shown in Supplementary Fig. 5. Herein, we observed that male participants less often believed in the accuracy of the PCR test (92.5%, 95% CI 90.2–94.3) and more often in the accuracy of the RAT (46.4%, 95% CI 42.5–50.4) as well as in the methods being indifferent (21.3%, 95% CI 18.3–24.7) compared to females (95.2%, 95% CI 90.2–94.3, *p, 38.1%, 95% CI 34.7–41.6, **p, and 16.7%, 95% CI 14.2–19.5, *p, respectively) and in multivariate statistical analysis (*p, **p, *p, Supplementary Fig. 5B).

Participants were asked to estimate the diagnostic sensitivity of both the PCR and the RAT for the detection of acute COVID-19. The median estimated sensitivity of the PCR was at 95% (IQR 90–98), and of the RAT significantly lower at 70% (IQR 58–85, ****p). Participants with a lower school diploma as their highest degree of education estimated the sensitivity of both assays significantly lower (median PCR: 90%, IQR 78–97), median RAT: 60%, IQR 35–75) compared to individuals with a bachelor’s, master’s or a higher academic degree and those holding a high school diploma (*p–***p for PCR, and **p–****p for RAT) as well as in multivariate analysis (***p, ****p, Fig. 3D). People who answered the questionnaire in a language other than German estimated the sensitivity of the RAT higher (median 80%, IQR 63–90) compared to German speakers (median 70%, IQR 58–85, **p, Supplementary Fig. 6A). Males predicted the sensitivity of both tests to be higher (median PCR 95%, IQR 90–99, median RAT 73%, IQR 60–88) than females (median PCR 94%, IQR 89–98, **p, median RAT 70%, IQR 55–82, ***p, Supplementary Fig. 6B). No differences in estimating the sensitivity of SARS-CoV-2 detection assays were observed between HCWs and non-healthcare professionals (Supplementary Fig. 6C). However, non-vaccinated individuals believed the sensitivity of the PCR test to be significantly lower (median 92%, IQR 80–97) than vaccinated participants (median 95%, IQR 90–98, ***p). and in multivariate analysis (****p, Supplementary Fig. 6D). Furthermore, there was a trend towards participants in younger age groups to estimate the sensitivity of both assays higher than individuals of older age (Supplementary Fig. 6E).

Next, we wondered which of the two COVID-19 detection assays were preferred. Interestingly, a larger fraction of participants agreed (fully or partially) to prefer the RAT (59.1%, 821/1388) compared to the PCR test (51.1%, 709/1388). Multivariate statistical analysis revealed that HCWs showed an adverse trend for preferring the PCR test more frequently (70.1%, 95% CI 63.1–76.3) than the RAT (42.4%, 95% CI 35.5–49.6, both (****p, Fig. 3F). Non-vaccinated individuals indicated strikingly less frequently to prefer the PCR test (23.7%, 95% CI 17.0–32.2) compared to vaccinated persons (53.6%, 95% CI 50.9–56.3, ****p) as well as in multivariate analysis (****p), and in turn indicated more often to prefer the RAT (78.8%, 95% CI 70.6–85.2) in multivariate analysis (****p, Fig. 3G). Similarly, there was a trend towards younger participants, to show more reluctance for the PCR and stronger preference for the RAT compared to older individuals, which was significant in multivariate analysis for the youngest age group (18–35 years, **p, and *p, respectively). Additional subgroup analyses revealed that non-German speakers and females significantly more often reported to prefer the PCR test (69.9%, 95% CI 58.6–79.2, and 54.0%, 95% CI 50.4–57.5) compared to German speakers (50.0%, 95% CI 47.3–52.7, **p) and males (47.2%, 95% CI 43.3–51.2, *p), respectively, as well as in multivariate analysis (****p, *p, Supplementary Fig. 7A, B). No statistically significant differences for the preference of any SARS-CoV-2 test were observed between participants with different educational backgrounds (Supplementary Fig. 7D).

### Behavior after receiving a SARS-CoV-2 test result

**Fig. 4:**
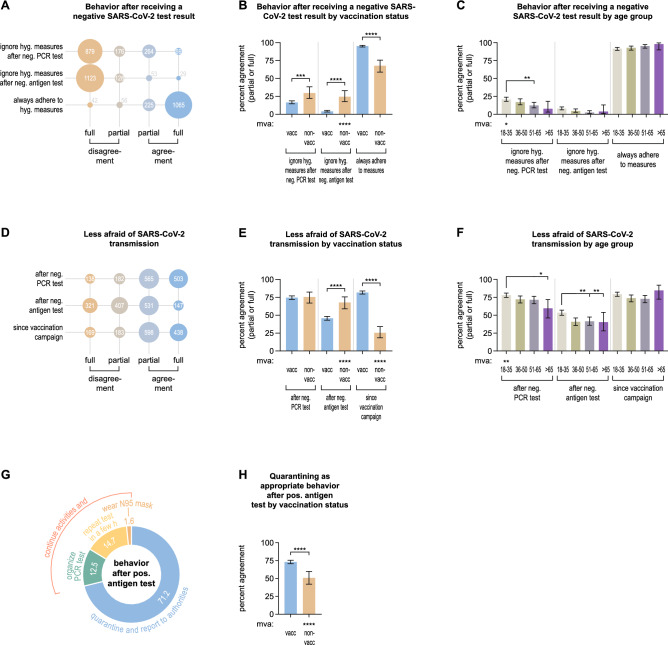
Behavior after receiving a SARS-CoV-2 test result. **A** Agreement and disagreement (partial and full) on being willing to ignore hygiene measures shortly after receiving a negative PCR or RAT result and the statement that it is always necessary to adhere to COVID-19 specific hygiene measures regardless of testing among all 1388 participants. **B**, **C** Percentages of all participants agreeing (partially or fully) to being willing to ignore hygiene measures after receiving a negative test result or to adhere to hygiene measures, stratified by vaccination status (**B**) and age group (**C**). **D** Agreement and disagreement (partial and full) for being less afraid of SARS-CoV-2 transmission after receiving a negative PCR or RAT result specifically and, in general, since the beginning of the vaccination campaign. E, F Percentages of all participants agreeing (partially or fully) to being less afraid after receiving a negative test result or, in general, since the beginning of the vaccination campaign by vaccination status (E), and age group (F). G Participants’ responses on what they believe is the appropriate behavior after receiving a positive RAT. Data is shown as percentages of all 1388 participants. H Percentages of all participants agreeing that quarantining is the appropriate behavior after receiving a positive RAT by vaccination status. Absolute numbers of participants giving certain answers are displayed in (A, D). Error bars in (B, C, E, F, H) indicate 95% confidence intervals. Asterisks above brackets indicate statistical significance between groups calculated with Fisher’s exact test (B, E, H), and Fisher’s exact test with Holm’s testing correction (C, F). Asterisks next to “mva” indicate statistical significance in multivariate analysis. If no asterisks are given, no statistical significance was detected. h—hours, hyg.—hygiene, mva—multivariate analysis, neg.—negative, non-vacc—non-vaccinated, pos.—positive, vacc—vaccinated

We asked the participants whether they thought it was no longer necessary to strictly adhere to pandemic-associated hygiene measures e.g., wearing a mask, social distancing, and hand disinfection, after receiving a negative COVID-19 test result. 24.0% (333/1388) of all individuals agreed (partially or fully) to be willing to ignore these hygiene measures after receiving a negative PCR test, but only 5.9% (82/1388) after receiving a negative RAT (Fig. 4A). On the contrary, 92.9% (1290/1388) agreed that hygiene measures should always be adhered to, irrespective of the test result (Fig. 4A). Remarkably, non-vaccinated individuals were more frequently willing to abandon hygiene measures after receiving a negative PCR (29.7%, 95% CI 22.2–38.4) or RAT (24.6%, 95% CI 17.7–33.1) and agreed significantly less often to the need to always adhere to hygiene measures (67.8%, 95% CI 58.9–75.6) compared to vaccinated persons (16.5%, 95% CI 14.6–18.7, ****p, 4.1%, 95% CI 3.2–5.4, ****p, and 95.3%, 95% CI 94.0–96.3, ****p, respectively, Fig. 4B). Furthermore, there was a trend towards participants from younger age groups to be more often willing to abandon hygiene measures after receiving a negative PCR test, which was significant in multivariate analysis for individuals between 18 and 35 years of age (*p, Fig. 4C). Additional subgroup analyses (Supplementary Fig. 8) revealed that females agreed more often to the need for always adhering to hygiene measures (94.4%, 95% CI 92.5–95.8) compared to males (91.2%, 95% CI 88.7–93.2, *p) and in multivariate analysis (**p, Supplementary Fig. 8B).

Subsequently, participants were asked whether they were less afraid of SARS-CoV-2 transmission to others in the 48 h after receiving a negative COVID-19 test result. 76.9% (1068/1388) agreed (fully or partially) to be less afraid of transmitting SARS-CoV-2 after receiving a negative PCR result, whereas only 48.8% (678/1388) reported less fear after a negative RAT result (Fig. 4D). Additionally, 74.6% (1036/1388) of all participants indicated to be less afraid of SARS-CoV-2 transmission since the beginning of the COVID-19 vaccination campaign (Fig. 4D). A significantly larger fraction of non-vaccinated individuals reported less fear of virus transmission after receiving a negative RAT (67.8%, 95% CI 58.9–75.6) than vaccinated persons (45.7%, 95% CI 42.9–48.4, ****p) and in multivariate analysis (****p, Fig. 4E). Also, non-vaccinated individuals agreed less often to feel safer since the beginning of the vaccination campaign (25.4%, 95% CI 18.4–34.0) compared to vaccinated persons (81.7%, 95% CI 79.5–83.8, ****p) and in multivariate statistical analysis (****p, Fig. 4E). We observed that younger participants were more confident to not transmit SARS-CoV-2 shortly after receiving a negative PCR test than people of older age, which was significant in multivariate analysis for the youngest age group (**p, Fig. 4F). Further subgroup analyses (Supplementary Fig. 9) showed that females were less afraid of virus transmission after a negative PCR test (77.6%, 95% CI 74.5–80.4) than males (70.7%, 95% CI 67.0–74.1, **p, Supplementary Fig. 9B), and that healthcare professionals were more afraid after a negative RAT (38.6%, 95% CI 31.9–45.8) compared to others (48.9%, 95% CI 46.1–51.7, *p) and in multivariate statistical analysis (*p, Supplementary Fig. 9C).

Finally, we asked about the participants’ thoughts on the correct behavior after receiving a positive RAT. 71.2% (988/1388) answered that they believed isolation and reporting to the responsible health authority was appropriate in that case, whereas 28.8% (400/1388) did not see the need to do so (Fig. 4G). 43.5% (174/400) of the latter agreed on organizing a PCR test, 51.0% (204/400) on repeating the RAT in a few hours and 5.5% (22/400) on simply wearing a N95/FFP2 mask. Further analyses revealed no significant differences between subgroups regarding the behavior after receiving a positive test result (Supplementary Fig. 10), except for non-vaccinated individuals, who substantially less frequently indicated that isolation is the appropriate behavior (50.8%, 95% CI 41.9–59.7) compared to vaccinated people (73.1%, 95% CI 70.6–75.4, ****p) and in multivariate analysis (****p, Fig. 4H).

## Discussion

In this cross-sectional study, in which approximately 0.1% of Munich’s population participated, we obtained profound insights into the perception of the two most common SARS-CoV-2 detection methods, which media were utilized by participants for gathering information, and their behavior after receiving negative or positive COVID-19 test results.

In a country like Germany, where both the PCR and the RAT are commonly accepted in COVID-19 surveillance and the adherence to hygiene measures heavily relies on self-motivation and self-responsibility, information sources and media play a crucial role [20, 21]. However, reliable data on which media sources are used by the public for information on COVID-19 testing are scarce. Our results show that online media as well as state and federal authorities were the most frequently used sources, whereas classical media, including television, radio, and newspapers, as well as social media were less relied on, in line with a study assessing media preferences for general information on COVID-19 among the German public [22]. This highlights, on one hand, the importance of governmental institutions for generating awareness and spreading knowledge on SARS-CoV-2 testing and, on the other hand, the potential dangers of dispersing misinformation via politicized or conspiracy theory-driven online media [23–25].

Albeit showing higher trust in the accuracy of the PCR test and estimating its sensitivity significantly higher, participants by trend preferred using RATs for COVID-19 testing. Due to its short turn-around time, RATs are more convenient to use, potentially explaining their preferability among participants. Furthermore, the possibility to use RATs in self-testing might lower the threshold to use them for those who are afraid of stigma [26], but conversely might negatively impact reporting to health authorities and thus compromise COVID-19 surveillance. Of note, both the RAT and the PCR test were available for free at test centers at the time the study was conducted.

Most individuals agreed that it was necessary to always adhere to COVID-19-specific hygiene measures independent of receiving a negative SARS-CoV-2 test result, indicating a general understanding of SARS-CoV-2 pathogenesis and spread as well as the meaningfulness of testing. However, a larger fraction of participants reported to be less afraid of viral transmission, in general, after the beginning of the vaccination campaign and, specifically, after receiving a negative PCR result or, to lesser extent, negative RAT result. Regarding receiving a positive RAT result, most individuals agreed that self-isolation was the correct response, albeit there being a substantial faction that disagreed to this sentiment. When self-testing is highly common, this behavior might lead to a considerable number of non-reported, non-quarantining COVID-19 cases, and thus potentially increases undetected disease spread. This in fact characterizes the situation in Germany in the fall of 2022.

Subgroup analyses revealed that especially non-vaccinated participants answered the questionnaire different from others. Non-vaccinated individuals rejected state and federal authorities more frequently as information sources and relied more on social media, which has been indicated to promote misinformation on COVID-19 as well as rumors, stigma, and conspiracy theories [27]. They disbelieved in the accuracy of the PCR test and displayed a strong preference for using RATs. Favoring RATs as a detection method with comparably low sensitivity might lead to higher numbers of non-detected SARS-CoV-2 infections in this subgroup [11–13, 16]. Non-vaccinated participants indicated more frequently to be willing to abandon hygiene measures, in general, as well as after receiving a negative SARS-CoV-2 test result, and less afraid of SARS-CoV-2 transmission after a negative RAT result. Finally, non-vaccinated individuals agreed less frequently to quarantining being the appropriate behavior after a positive RAT. Taken together, our findings indicate that this subgroup displays a higher risk for SARS-CoV-2 infection and disease spread. This is striking, because non-vaccinated individuals are in general more susceptible to SARS-CoV-2 infection and severe COVID-19 [2–4], and thus should be well informed about test performance and hygiene measures.

Other than using online and social media more frequently, younger participants showed a preference for RATs and were less afraid of SARS-CoV-2 transmission after receiving negative test results, potentially making this subgroup more vulnerable to viral infection. Interestingly, individuals with lower school diplomas as their highest educational degree, gathered information less often from online media and scientific sources and, at the same time, reported more often to be uninformed, possibly explaining why this subgroup estimated the sensitivity of both SARS-CoV-2 detection methods lower than others. In contrast to others, HCWs preferred the PCR test over RATs for COVID-19 testing. This might be due to weekly PCR tests for all patient care employees at the study site hospitals, the LMU Klinikum, that made HCWs more accustomed to this testing method.

A limitation of this study is that the study population might not be fully representative of Munich’s population. Compared to the city’s age demographic [19], a larger fraction of young individuals and less elderly participated in the study, which could be caused by using an online questionnaire that was to be accessed with a smartphone. Individuals with low education level and non-German speakers were underrepresented in the study and, in contrast, HCWs were overrepresented. This is potentially due to recruiting participants, among others, at the LMU and the university hospital and giving out the recruitment material mainly in German language. Furthermore, vaccination rates among study participants were higher than estimated for Munich at the time the study was conducted [28], which could be because of non-vaccinated individuals being reluctant to participate in research related to COVID-19 and due to overrepresentation of HCWs in the study. On a more general level, this study likely is biased towards self-reporting, well-educated, health conscious vaccinated German speakers with a positive attitude towards health.

In conclusion, our study, for the first time, gives in-depth insights into the perception of SARS-CoV-2 detection methods, media utilized for gathering information, and behaviors after receiving negative or positive test results among urban citizens. It highlights the importance of state and federal authorities and online media for informing the public about testing, shows that RATs are often preferred even though the PCR test is more trusted, and that most people display cautious behavior to avoid infection and spread of SARS-CoV-2. Strikingly, non-vaccinated individuals frequently displayed divergent believes and behaviors that possibly make them more prone to viral infection and spread. Even though this is a regional study, its findings might be broadly applicable and, thus, helpful for conceiving and conducting future public health information and surveillance campaigns.

## Supplementary Information

Below is the link to the electronic supplementary material.

Supplementary file1 (PDF 3848 KB)

## Data Availability

The data used and/or analyzed during the current study are available from the corresponding author on reasonable request.
