# Supplementary material for: Vaccine-hesitant individuals accumulate additional COVID-19 risk due to divergent perception and behaviors related to SARS-CoV-2 testing: a population-based, cross-sectional study
Source: Infection. 2022 Nov 10;51(4):909–19. doi: 10.1007/s15010-022-01947-z (PMC9647754; doi:10.1007/s15010-022-01947-z)
Supplement: Supplementary file 1 — Supplementary file1 (PDF 3848 KB) [file 15010_2022_1947_MOESM1_ESM.pdf]

Supplementary Information

Vaccine-hesitant individuals accumulate additional COVID-19 risk due to divergent perception and behaviors related to SARS-CoV-2 testing – a population based, cross-sectional study

Paul R. Wratil, Katharina Kotter, Marie L. Bischof, Sophie Hollerbach, Elif Apak, Anna-Lena Kalteis, Tamara Nayeli-Pflantz, Lars Kaderali, Kristina Adorjan, Oliver T. Keppler

Corresponding Authors:

Paul R. Wratil (wratil@mvp.lmu.de)

Kristina Adorjan (kristina.adorjan@med.uni-muenchen.de)

Oliver T. Keppler (keppler@mvp.lmu.de)

Supplementary figure legends

Supplementary Fig. 1 Subgroup analysis of vaccination rates.

Percentages of all 1,388 Participants reporting to be vaccinated against COVID-19 by language used to answer the questionnaire (A), gender (B), healthcare worker status (C), age group (D), and highest degree of education (E). Error bars indicate 95 % confidence intervals. Asterisks above brackets indicate statistical significance between groups calculated using Fisher's exact test (A-C) or Fisher's exact test with Holm's testing correction (D, E). If no asterisks are given, no statistical significance was detected. compl. – completed, deg. – degree, dipl. – diploma, HCW – healthcare worker.

Supplementary Fig. 2 Subgroup analysis of reluctance towards vaccination.

Percentages of all 1,388 Participants reporting to be unwilling or uncertain to become vaccinated against COVID-19 by language used to answer the questionnaire (A), gender (B), healthcare worker status (C), age group (D), and highest degree of education (E). Error bars indicate 95 % confidence intervals. Statistical significance between groups was

tested using Fisher's exact test (A-C) or Fisher's exact test with Holm's testing correction (D, E) but no significant differences were detected. compl. – completed, deg. – degree, dipl. – diploma, HCW – healthcare worker.

Supplementary Fig. 3 Subgroup analysis of reasons for reluctance towards COVID-19 vaccination.

Percentages of agreement (full or partial) on reasons against vaccination among those 97 Participants reporting to be unwilling or uncertain to become vaccinated against COVID-19 by language used to answer the questionnaire (A), gender (B), healthcare worker status (C), age group (D), and highest degree of education (E). Error bars indicate 95 % confidence intervals. Asterisks next to brackets indicate statistical significance between groups calculated using Fisher's exact test (A-C) or Fisher's exact test with Holm's testing correction (D, E). If no asterisks are given, no statistical significance was detected.

Supplementary Fig. 4 Additional subgroup analyses on preferred information sources on SARS-CoV-2 testing.

Percentages of all 1,388 participants agreeing (partially or fully) on gathering information on testing using different sources by gender (A), language used to answer the questionnaire (B), highest degree of education (C), and healthcare worker status (D). Error bars indicate 95 % confidence intervals. Asterisks next to brackets indicate statistical significance between groups calculated using Fisher's exact test (A, B, D) or Fisher's exact test with Holm's testing correction (C). Asterisks below "mva" indicate statistical significance in multivariate analysis. If no asterisks are given, no statistical significance was detected. mva – multivariate analysis.

Supplementary Fig. 5 Additional subgroup analyses on the belief in the accuracy of different SARS-CoV-2 detection methods.

Percentages of all 1,388 participants agreeing (partially or fully) on believing in the accuracy of the PCR and RAT and the statement of being unaware of differences between the two SARS-CoV-2 detection methods by language used to answer the questionnaire (A), gender (B), healthcare worker status (C), age group (D), and highest level of education (E). Error bars indicate 95 % confidence intervals. Asterisks above brackets indicate statistical significance between groups calculated using Fisher's exact test (A-C) or Fisher's exact test with Holm's testing correction (D, E). Asterisks next to "mva" indicate statistical significance in multivariate analysis. If no asterisks are given, no statistical significance was detected. compl. – completed, deg. – degree, dipl. – diploma, HCW – healthcare worker, mva – multivariate

analysis.

Supplementary Fig. 6 Additional subgroup analyses on the estimated sensitivities of different SARS-CoV-2 detection methods.

Comparison of the estimated sensitivities of the PCR and the rapid antigen test among all 1,388 participants by language used to answer the questionnaire (A), gender (B), healthcare worker status (C), vaccination status (D), and age group (E). Box plots depict medians, bounds between upper and lower quartiles, and whiskers between the 10th and 90th percentile. Asterisks above brackets indicate statistical significance between groups calculated with Wilcoxon rank sum test with continuity correction. Asterisks next to “mva” indicate statistical significance in multivariate analysis. If no asterisks are given, no statistical significance was detected. HCW – healthcare worker, mva – multivariate analysis.

Supplementary Fig. 7 Additional subgroup analyses on preferred SARS-CoV-2 detection methods.

Percentages of all 1,388 participants agreeing (partially or fully) on preferring PCR and RATs for SARS-CoV-2 testing by language used to answer the questionnaire (A), gender (B), and highest level of education (E). Error bars indicate 95 % confidence intervals. Asterisks above brackets indicate statistical significance between groups calculated using Fisher’s exact test (A, B) or Fisher’s exact test with Holm’s testing correction (C). Asterisks next to “mva” indicate statistical significance in multivariate analysis. If no asterisks are given, no statistical significance was detected. compl. – completed, deg. – degree, dipl. – diploma, mva – multivariate analysis.

Supplementary Fig. 8 Additional subgroup analyses on behaviors after receiving a negative test.

Agreement (partial or full) on being willing to ignore hygiene measures shortly after receiving a negative PCR or RAT result and the statement that it is always necessary to adhere to COVID-19 specific hygiene measures regardless of testing among all 1,388 participants by language used to answer the questionnaire (A), gender (B), healthcare worker status (C), and highest degree of education (D). Asterisks above brackets indicate statistical significance between groups calculated using Fisher’s exact test (A-C) or Fisher’s exact test with Holm’s testing correction (D). Asterisks next to “mva” indicate statistical significance in multivariate analysis. If no asterisks are given, no statistical significance was detected. compl. – completed, deg. – degree, dipl. – diploma, HCW – healthcare worker, mva – multivariate

analysis, neg. – negative.

Supplementary Fig. 9 Additional subgroup analyses on fear of viral transmission.

Agreement (partial or full) on being less afraid after receiving a negative test result or, in general, since the beginning of the vaccination campaign among all 1,388 participants by language used to answer the questionnaire (A), gender (B), healthcare worker status (C), and highest degree of education (D). Asterisks above brackets indicate statistical significance between groups calculated using Fisher's exact test (A-C) or Fisher's exact test with Holm's testing correction (D). Asterisks next to "mva" indicate statistical significance in multivariate analysis. If no asterisks are given, no statistical significance was detected. compl. – completed, deg. – degree, dipl. – diploma, HCW – healthcare worker, mva – multivariate analysis, neg. – negative.

Supplementary Fig. 10 Additional subgroup analyses on quarantining after receiving a positive rapid antigen test.

Percentages of all 1,388 participants agreeing that quarantining is the appropriate behavior after receiving a positive RAT by language used to answer the questionnaire (A), gender (B), healthcare worker status (C), age group (D), and highest degree of education (E). Error bars indicate 95 % confidence intervals. Statistical significance was tested between groups using Fisher's exact test (A-C) or Fisher's exact test with Holm's testing correction (D, E), and in multivariate analysis, but no significant differences were detected. compl. – completed, deg. – degree, dipl. – diploma, HCW – healthcare worker, mva – multivariate analysis.

Supplementary Figure S1

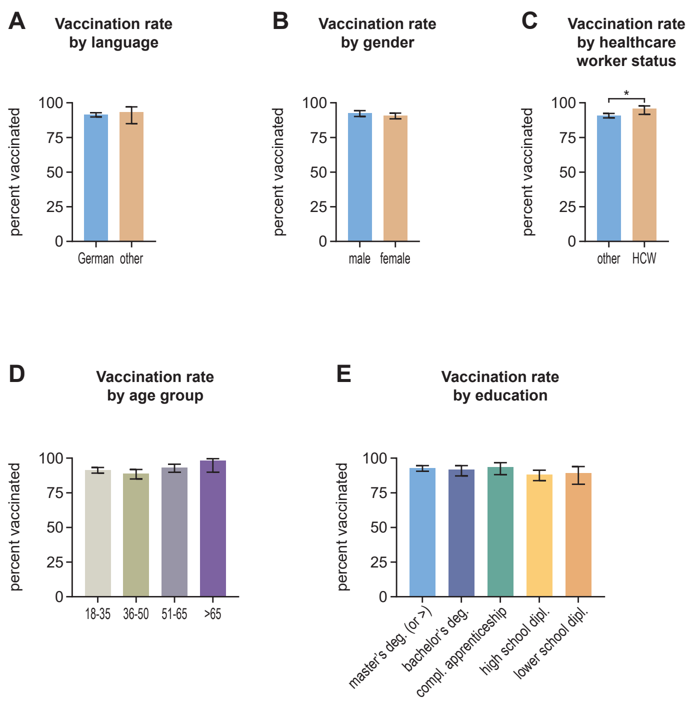

Supplementary Figure S2

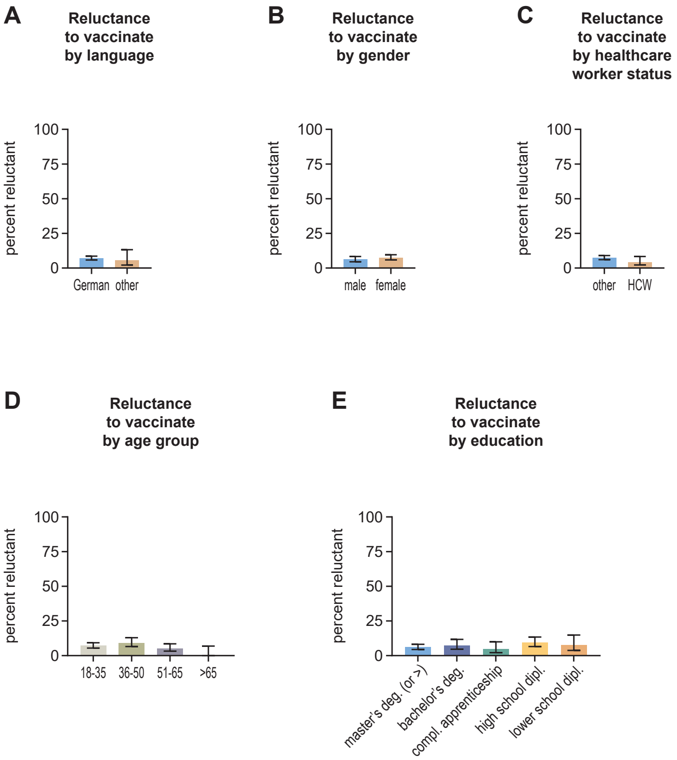

Supplementary Figure S3

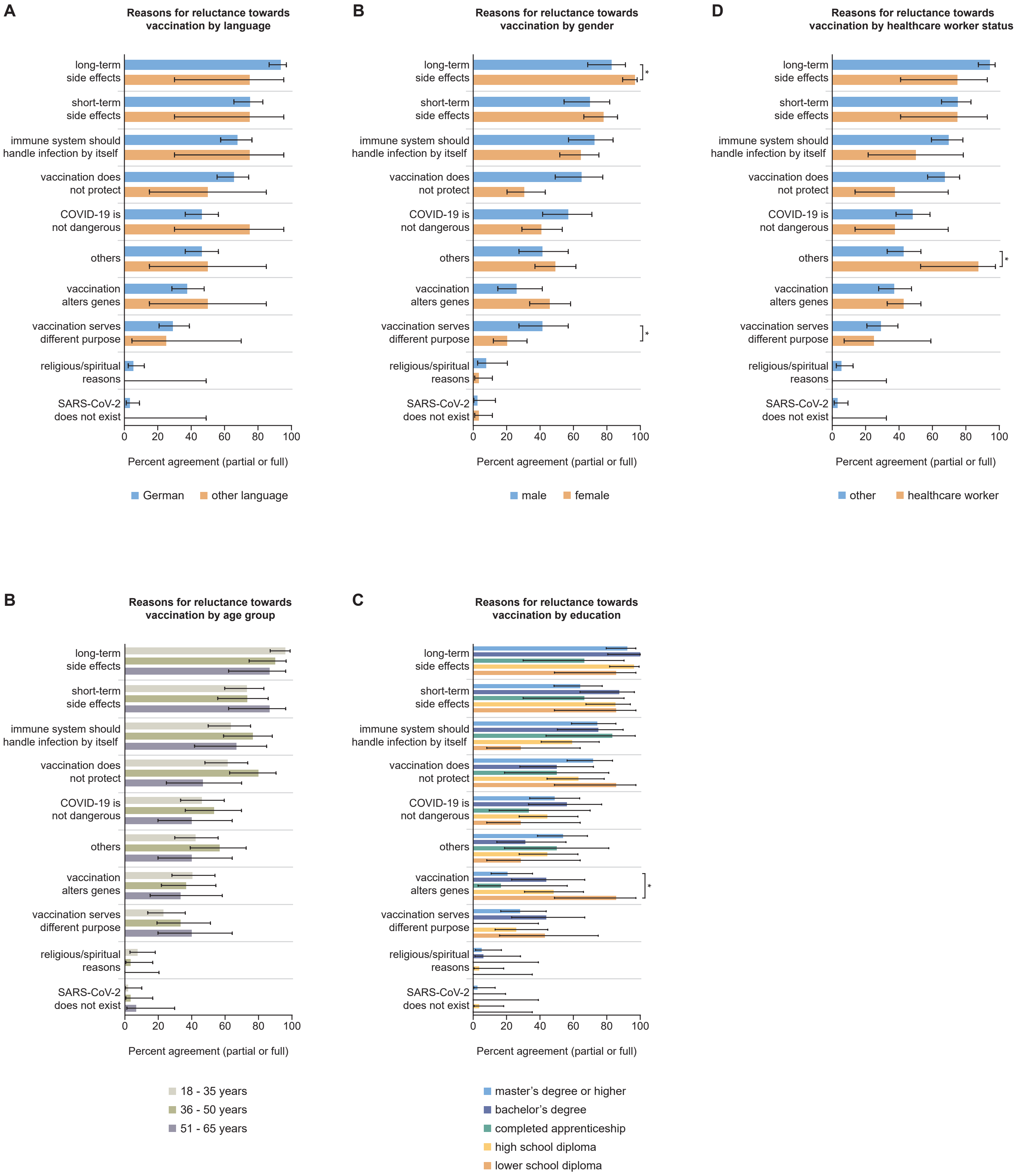

Supplementary Figure S4

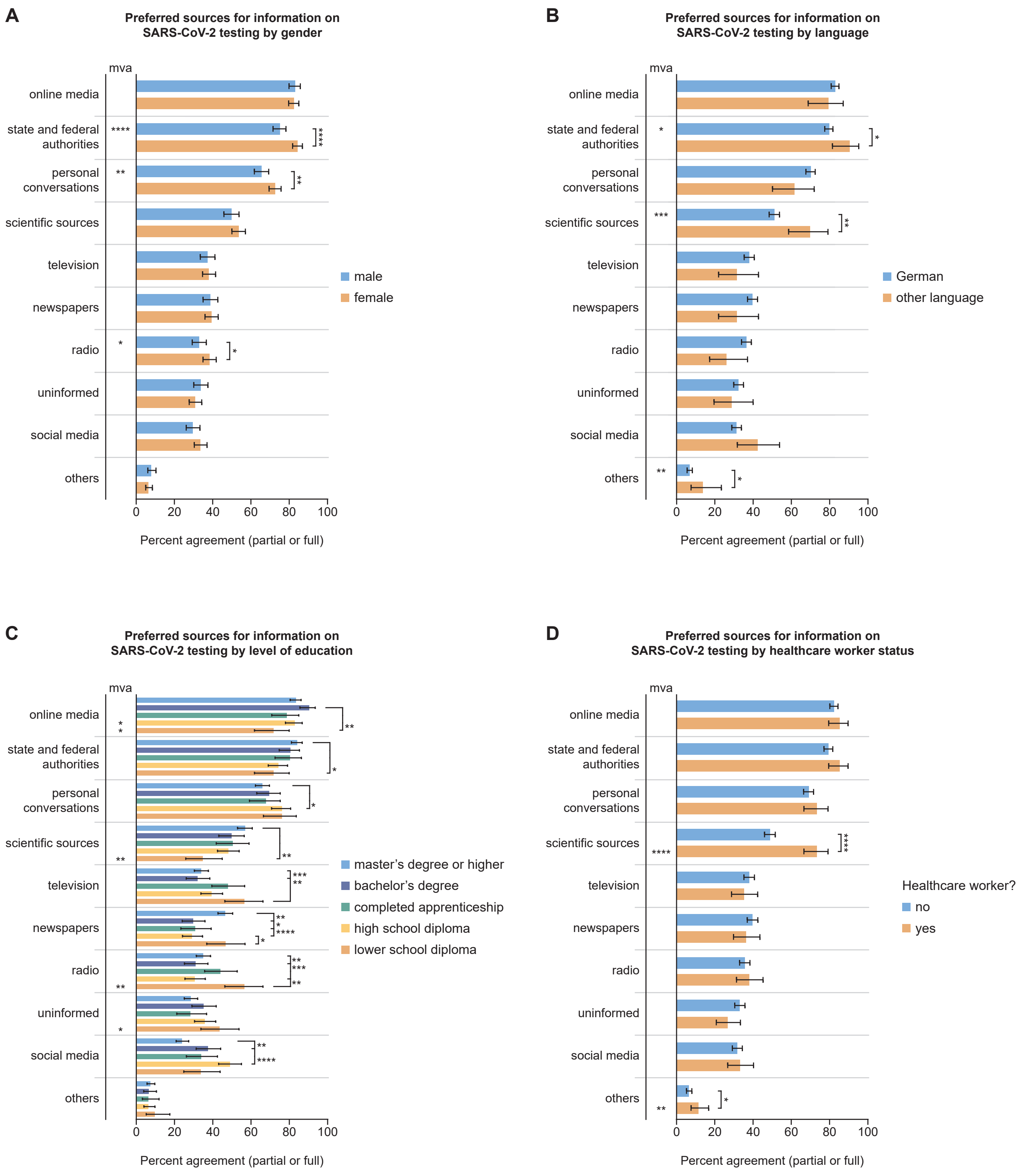

Supplementary Figure S5

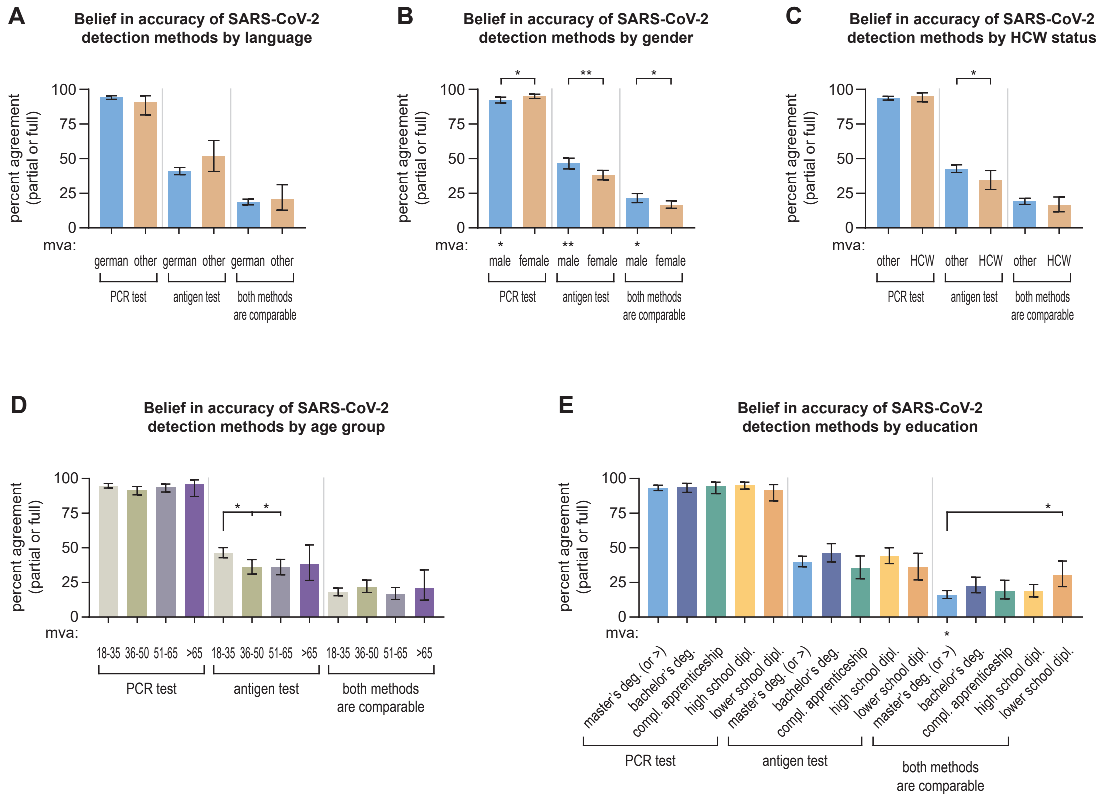

Supplementary Figure S6

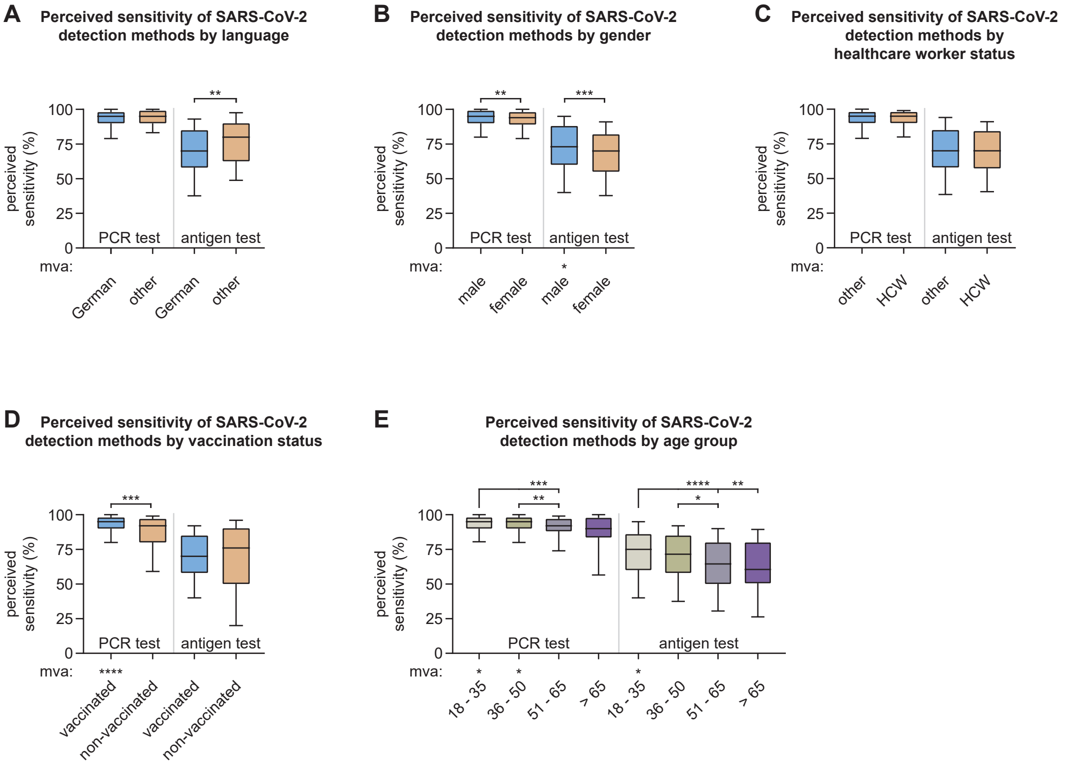

Supplementary Figure S7

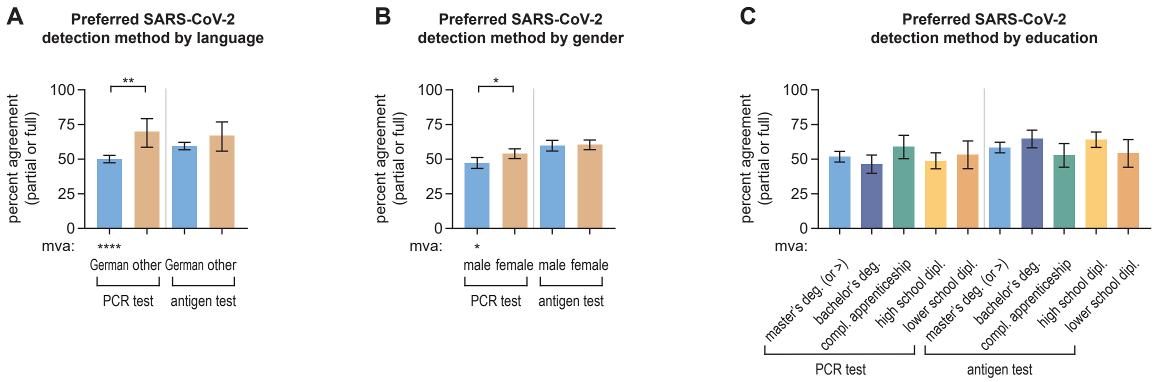

Supplementary Figure S8

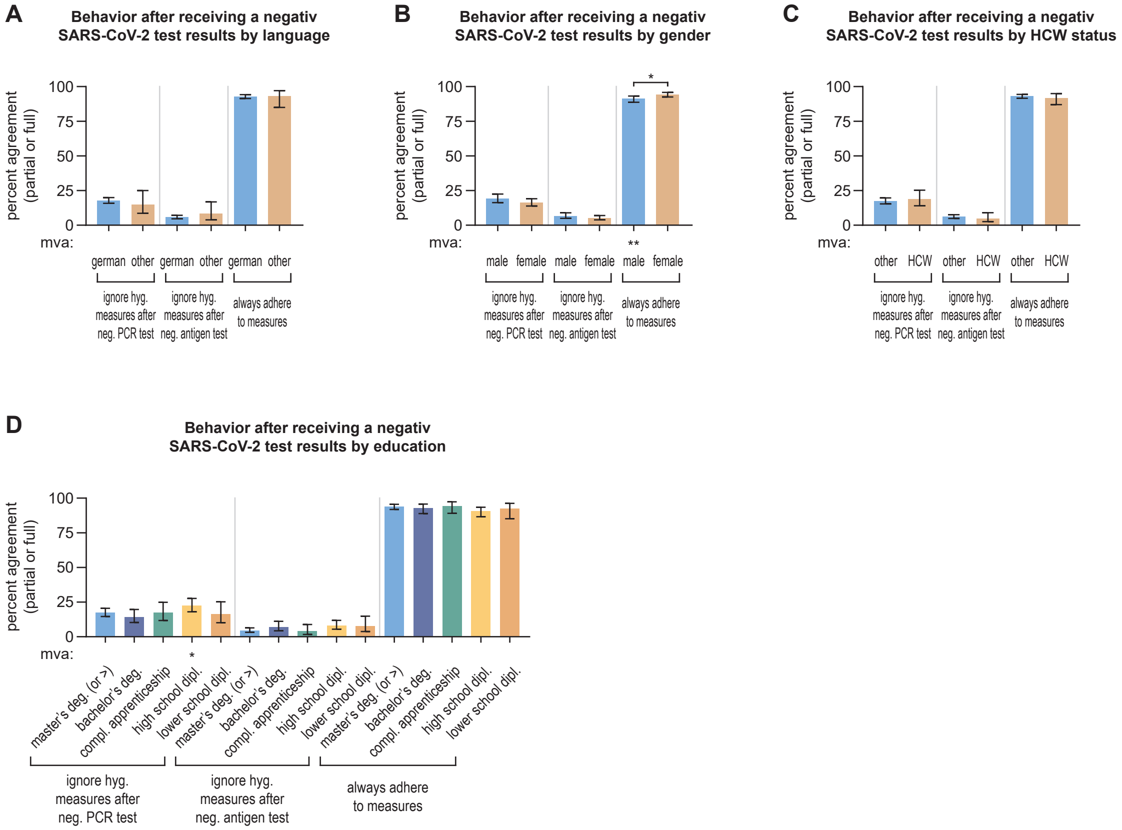

Supplementary Figure S9

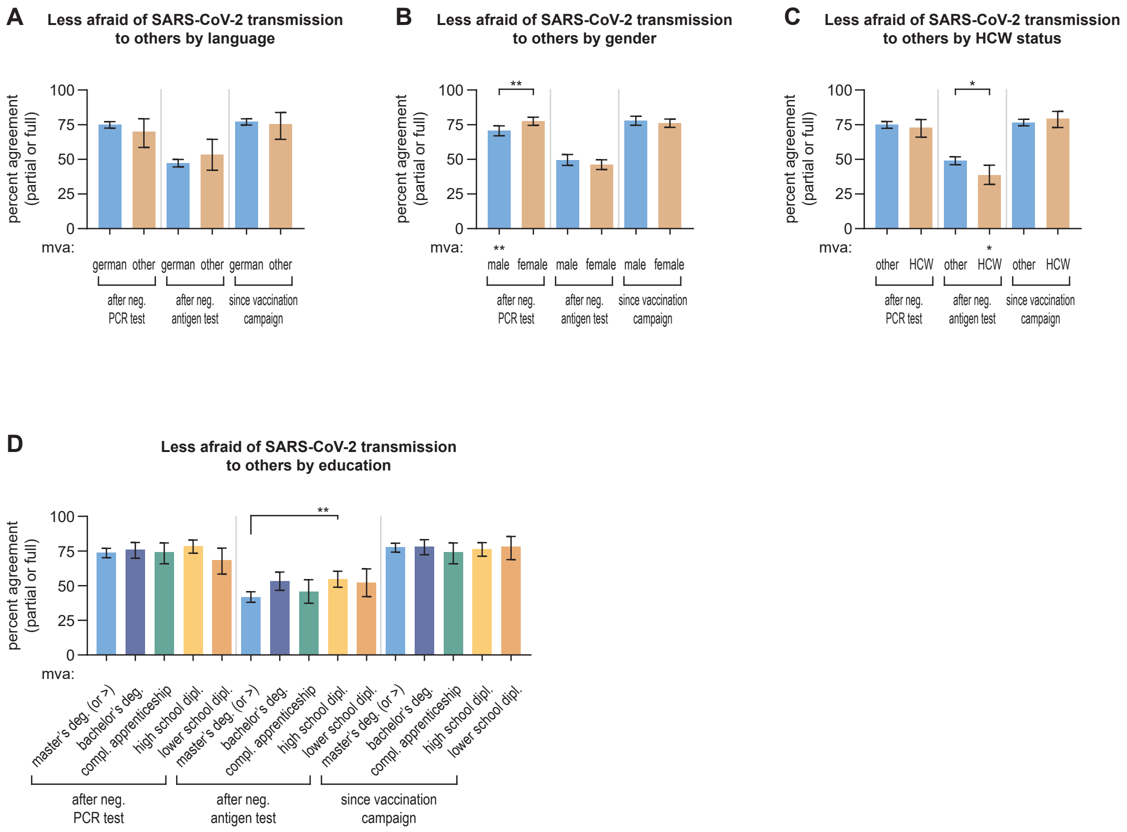

Supplementary Figure S10

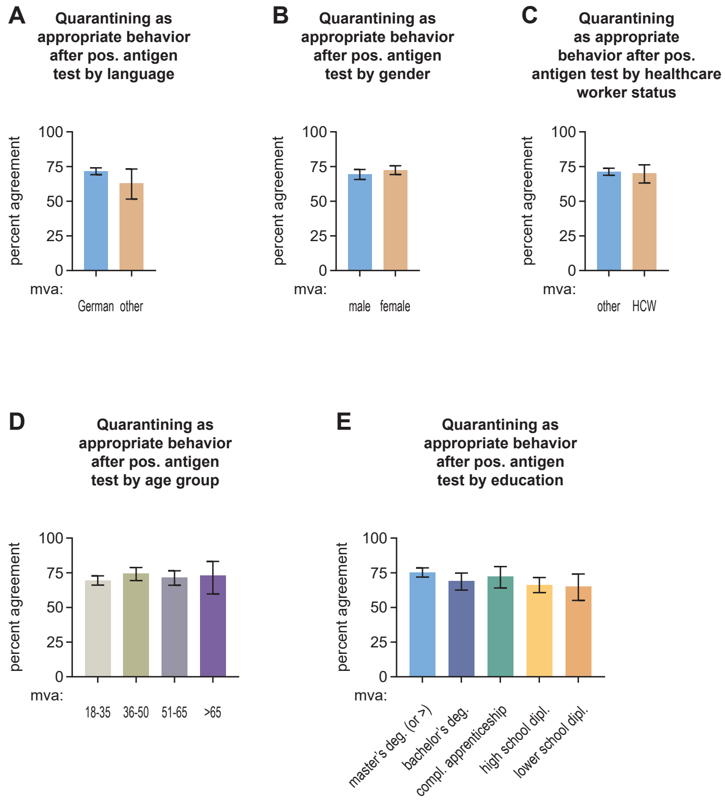

Supplementary tables

Supplementary Table 1. Study questionnaire in English and participants' answers.

**Language used to answer questionnaire**	Pos/total	(%)
German	1,315/1,388	(94.7)
English	52/1,388	(3.75)
Spanish	5/1,388	(0.36)
Italian	13/1,388	(0.94)
Turkish	3/1,388	(0.22)
**Gender**	Pos/total	(%)
female	767/1,388	(55.3)
male	614/1,388	(44.2)
other	7/1,388	(0.5)
**Age**		
**Median (95% CI): 34 (33-36)**	Pos/total	(%)
18-35	728/1,388	(52.4)
36-50	324/1,388	(23.3)
51-65	284/1,388	(20.5)
>65	52/1,388	(3.75)

Which type of media do you use most often to inform yourself about SARS-CoV-2 testing?								
	I agree.		I partially agree.		I partially disagree.		I disagree.	
	Pos/total	(%)	Pos/total	(%)	Pos/total	(%)	Pos/total	(%)
Television	213/1,388	(15.35)	309/1,388	(22.26)	354/1,388	(25.50)	512/1,388	(36.89)
Daily or weekly newspapers	224/1,388	(16.14)	321/1,388	(23.13)	282/1,388	(20.32)	561/1,388	(40.42)
Online media (e.g., websites, podcasts)	734/1,388	(52.88)	415/1,388	(29.90)	120/1,388	(8.65)	119/1,388	(8.57)
Radio	174/1,388	(12.54)	325/1,388	(23.41)	293/1,388	(21.11)	596/1,388	(42.94)
Social networks (e.g., Facebook, Twitter, Instagram, YouTube, TikTok)	196/1,388	(14.12)	247/1,388	(17.80)	261/1,388	(18.80)	684/1,388	(49.28)
Scientific sources (e.g., scientific articles, clinical trial reports)	306/1,388	(22.05)	417/1,388	(30.04)	342/1,388	(24.64)	323/1,388	(23.27)
Information from state and federal authorities (e.g., Paul-Ehrlich-Institut, Robert Koch-Institut, WHO, CDC)	540/1,388	(38.90)	574/1,388	(41.35)	167/1,388	(12.03)	107/1,388	(7.71)
Personal conversations with other people	368/1,388	(26.51)	599/1,388	(43.16)	311/1,388	(22.41)	110/1,388	(7.93)
I do not specifically inform myself about testing.	148/1,388	(10.66)	299/1,388	(21.54)	368/1,388	(26.51)	573/1,388	(41.28)
Other media	52/1,388	(3.75)	47/1,388	(3.39)	213/1,388	(15.35)	1,076/1,388	(77.52)

What do you think about the differences between rapid antigen testing and PCR testing for detection of SARS-CoV-2 infection?								
	I agree.		I partially agree.		I partially disagree.		I disagree.	
	Pos/total	(%)	Pos/total	(%)	Pos/total	(%)	Pos/total	(%)
I don't know any differences between testing methods.	150/1,388	(10.81)	110/1,388	(7.93)	122/1,388	(8.79)	1,006/1,388	(72.48)
I am convinced of the accuracy of the rapid antigen tests.	78/1,388	(5.62)	499/1,388	(35.95)	582/1,388	(41.93)	229/1,388	(16.50)
I am convinced of the accuracy of the PCR tests.	891/1,388	(64.19)	413/1,388	(29.76)	54/1,388	(3.89)	30/1,388	(2.16)
I prefer rapid antigen tests because they provide the test result within 15-30 minutes.	332/1,388	(23.92)	499/1,388	(35.95)	368/1,388	(26.51)	189/1,388	(13.62)
I prefer PCR tests, even if they take longer.	382/1,388	(27.52)	327/1,388	(23.56)	452/1,388	(32.56)	227/1,388	(16.35)

If 100 individuals with acute SARS-CoV-2 infection were tested, in how many of those individuals would the following testing system actually confirm the infection?	
	Median (95% CI)
Antigen rapid test	70 (70-72)
PCR test	95 (95-95)

To what extent do you agree with the following statements?								
	I agree.		I partially agree.		I partially disagree.		I disagree.	
	Pos/total	(%)	Pos/total	(%)	Pos/total	(%)	Pos/total	(%)
After a negative rapid antigen test result, it is no longer necessary to strictly adhere to hygiene measures.	29/1,388	(2.09)	53/1,388	(3.82)	183/1,388	(13.18)	1,123/1,388	(80.91)
After a negative PCR result, it is no longer necessary to strictly adhere to the hygiene measures.	69/1,388	(4.97)	176/1,388	(12.68)	264/1,388	(19.02)	879/1,388	(63.33)
Regardless of the test result, hygiene measures should always be adhered to.	1,065/1,388	(76.73)	225/1,388	(16.21)	56/1,388	(4.03)	42/1,388	(3.03)

What do you think about the risk of infection with SARS-CoV-2?								
	I agree.		I partially agree.		I partially disagree.		I disagree.	
	Pos/total	(%)	Pos/total	(%)	Pos/total	(%)	Pos/total	(%)
In the next 48h after a negative rapid antigen test result, I have little fear of infecting others with SARS-CoV-2.	147/1,388	(10.59)	513/1,388	(36.96)	407/1,388	(29.32)	321/1,388	(23.13)
In the next 48h after a negative PCR test result, I have little fear of infecting others with SARS-CoV-2.	438/1,388	(31.56)	598/1,388	(43.08)	183/1,388	(13.18)	169/1,388	(12.18)
Since the start of the vaccination campaign, I am less afraid of the spread of SARS-CoV-2 than I was before.	503/1,388	(36.24)	565/1,388	(40.71)	182/1,388	(13.11)	138/1,388	(9.94)

Which of the following behaviors do you think is most appropriate after you received a positive result using the rapid antigen test?		
	Pos/total	(%)
I immediately quarantine at home and report to the health department and/or my family doctor.	988/1,388	(71.18)
I organize a PCR test and then continue my activities until the PCR test result is available.	174/1,388	(12.54)
I put on an N95/FFP2 mask and continue to work or visit my relatives regardless.	22/1,388	(1.59)
I repeat the test in a few hours because it could be false positive.	204/1,388	(14.70)

Have you been vaccinated against COVID-19?		
	Pos/total	(%)
Yes	1,270/1,388	(91.50)
No	118/1,388	(8.50)

Are you planning to get vaccinated against COVID-19?		
	Pos/total	(%)
Yes	21/188	(17.80)
Maybe	46/188	(38.98)
No	51/188	(43.22)

Why are you considering/planning not to get vaccinated against COVID-19?								
	I agree.		I partially agree.		I partially disagree.		I disagree.	
	Pos/total	(%)	Pos/total	(%)	Pos/total	(%)	Pos/total	(%)
I oppose the vaccination for religious/spiritual reasons.	3/97	(3.09)	2/97	(2.06)	7/97	(7.22)	85/97	(87.63)
I am afraid of short-term side effects.	36/97	(37.11)	13/97	(38.14)	11/97	(11.34)	13/97	(38.14)
I am afraid of long-term side effects.	61/97	(62.89)	29/97	(29.90)	5/97	(5.15)	2/97	(2.06)
I do not believe that the vaccination protects against SARS-CoV-2 infections.	29/97	(29.90)	34/97	(35.05)	23/97	(23.71)	11/97	(11.34)
I do not think COVID-19 is dangerous and therefore find the vaccination unnecessary.	16/97	(16.49)	30/97	(30.93)	25/97	(25.77)	26/97	(26.80)
I am afraid that the vaccination could change my genes.	15/97	(15.46)	22/97	(22.68)	25/97	(25.77)	35/97	(36.08)
I refuse the vaccination because I think that the body's immune system can and must deal with the pathogen itself.	20/97	(20.62)	46/97	(47.42)	18/97	(18.56)	13/97	(13.40)
I do not believe that SARS-CoV-2 exists and therefore find the vaccination unnecessary.	1/97	(1.03)	2/97	(2.06)	4/97	(4.12)	90/97	(92.78)
I fear that the vaccination actually serves another purpose than protection against a virus.	12/97	(12.37)	16/97	(16.49)	27/97	(27.84)	42/97	(43.30)
Other reasons	33/97	(34.02)	12/97	(12.37)	7/97	(7.22)	45/97	(46.39)

What is your highest level of education?		
	Pos/total	(%)
Completed apprenticeship (e.g. as an electrician, bank clerk, police officer)	127/1,388	(9.15)
Extended elementary school diploma	15/1,388	(1.08)
High school diploma	285/1,388	(20.53)
I have a different school/vocational qualification.	23/1,388	(1.66)
Middle school diploma	77/1,388	(5.55)
No school diploma	3/1,388	(0.22)
University degree - Bachelor	216/1,388	(15.56)
University degree - Master	437/1,388	(31.48)
University degree - PhD or higher	205/1,388	(14.77)

Are you a medical professional (e.g. doctor, nurse, physiotherapist)?		
	Pos/total	(%)
Yes	184/1,388	(13.26)
No	1,204/1,388	(86.74)
